# Generation of a Peptide Vaccine Candidate against Falciparum Placental Malaria Based on a Discontinuous Epitope

**DOI:** 10.3390/vaccines8030392

**Published:** 2020-07-18

**Authors:** Catherine J. Mitran, Lauren M. Higa, Michael F. Good, Stephanie K. Yanow

**Affiliations:** 1 School of Public Health, University of Alberta, Edmonton, AB T6G 2R3, Canada; mitran@ualberta.ca (C.J.M.); higa@ualberta.ca (L.M.H.); 2 Institute for Glycomics, Griffith University, Southport, Queensland 4215, Australia; michael.good@griffith.edu.au; 3 Department of Medical Microbiology & Immunology, Faculty of Medicine & Dentistry, University of Alberta, Edmonton, AB T6G 2R3, Canada

**Keywords:** *Plasmodium falciparum*, Plasmodium vivax, vaccine, placental malaria, peptide, PvDBP, VAR2CSA

## Abstract

In pregnant women, *Plasmodium falciparum-*infected red blood cells adhere to the placenta via the parasite protein VAR2CSA. Two vaccine candidates based on VAR2CSA are currently in clinical trials; however, these candidates failed to elicit strain-transcending antibody responses. We previously showed that a cross-reactive monoclonal antibody (3D10) raised against the *P. vivax* antigen PvDBP targets epitopes in VAR2CSA. We now aim to design a peptide vaccine against VAR2CSA based on the epitope that generated 3D10. We mapped the epitope to subdomain 1 (SD1) of PvDBP and identified a peptide that contained the minimal sequence. However, this peptide did not elicit cross-reactive VAR2CSA antibodies in mice. When tested against a broader, overlapping peptide array spanning SD1, 3D10 in fact recognized a discontinuous epitope consisting of three segments of SD1. These findings presented the challenge to generate this larger structural epitope as a synthetic peptide since it is stabilized by two pairs of disulfide bonds. We overcame this using a synthetic scaffold to conformationally constrain the SD1 peptide and coupled it to keyhole limpet hemocyanin (KLH). The SD1-KLH conjugate elicited antibodies in mice that cross-reacted with VAR2CSA. This strategy successfully recapitulated a discontinuous epitope with a synthetic peptide and represents the first heterologous vaccine candidate against VAR2CSA.

## 1. Introduction

Pregnancy-associated malaria caused by infection with the parasite *Plasmodium falciparum* can result in preterm birth, low birth weight babies, spontaneous abortion, and infant and maternal death [1]. Despite ongoing efforts to develop a vaccine to prevent malaria in pregnancy, no effective vaccine exists. The leading vaccine candidates are based on VAR2CSA, a *P. falciparum* protein that mediates sequestration of infected red blood cells to the placenta [2,3,4,5,6,7]. While these vaccines show promise by eliciting strong antibodies to the homologous VAR2CSA allele, they failed to elicit broadly neutralizing antibodies against heterogeneous parasite strains due to extensive natural polymorphisms within VAR2CSA [6,7,8]. Polyvalent vaccines that include multiple alleles of VAR2CSA or new vaccines that target conserved epitopes are urgently needed.

We discovered an alternate source of antibodies to VAR2CSA that can be exploited for vaccine design. The source of these antibodies is an epitope shared between the Duffy binding-like (DBL) domain of the *P. vivax* Duffy binding protein (PvDBP), an invasion protein expressed by *P. vivax* merozoites, and the DBL domains of VAR2CSA [9]. Despite sharing only 16–21% sequence homology, the DBL domains of VAR2CSA and PvDBP have shared epitopes that are targeted by cross-reactive antibodies elicited by natural exposure to *P. vivax* infection or through immunization with PvDBP [9,10]. A mouse monoclonal antibody (3D10 mAb) raised against the DBL domain of PvDBP recognized VAR2CSA and blocked parasite adhesion in an in vitro assay of placental malaria [9]. When we investigated the cross-reactive target of 3D10, we found that it recognized epitopes that were cryptic in VAR2CSA [10]. Given the cryptic nature of these epitopes, they are unlikely to be under the same immune pressure as the more immunodominant epitopes in the protein. Therefore, identifying and targeting these epitopes in VAR2CSA may present a viable vaccine strategy against malaria in pregnancy. Targeting cryptic or subdominant epitopes has also been employed in the development of vaccine candidates for group A streptococcus [11], Ebola [12], and influenza [13,14,15].

Here, we designed an epitope-focused vaccine candidate against VAR2CSA based on the epitope that generated the 3D10 mAb. This epitope has been localized to subdomain 1 (SD1) of PvDBP region II (DBPII) [10,16,17], and we used peptide arrays to refine the epitope to three discontinuous segments of SD1. Using a synthetic scaffold, we recapitulated this discontinuous epitope within a conformationally constrained peptide. Importantly, this peptide elicited antibodies in mice and a rabbit that recognized DBPII and cross-reacted with VAR2CSA. 

## 2. Materials and Methods 

### 2.1. Synthetic Peptide Design and Conjugation

**Figure 1:**
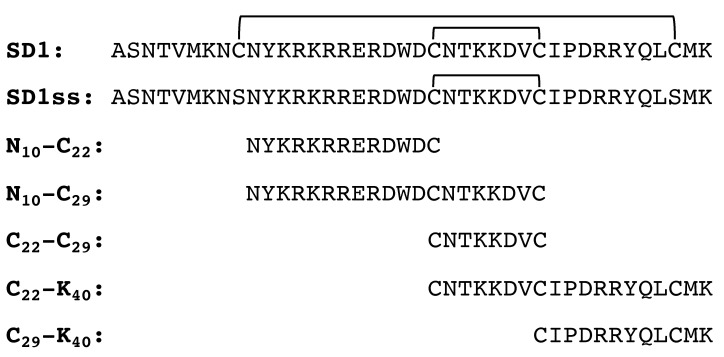
Synthetic peptides were designed to cover the subdomain 1 (SD1) region of DBPII. The subscript numbers indicate the amino acid position in the parent SD1 peptide. Disulfide bonds are noted with horizontal lines.

Peptides representing different regions of SD1 in DBPII (Figure 1) were synthesized (Synpeptides Co., Shanghai, China) based on the sequence from the Sal 1 allele of PvDBP. The SD1ss peptide was designed to cover the entire SD1 region, with one pair of cysteines (C_9_ and C_38_) mutated to serine to control disulfide bond formation. N_10_-C_22_ was conjugated to diphtheria toxoid (DT) using 6-maleimido-caproyl n-hydroxy succinimide (MCS) (Sigma, Oakville, Canada) [18]. Briefly, MCS dissolved in dimethylformamide (DMF) (33.3 mg/mL) was added to a solution of DT in 0.1 M phosphate buffer (10 mg/mL) and mixed slowly at room temperature for 1 h. The modified carrier protein was then dialyzed against 0.1 M phosphate buffer containing 0.1 M ethylenediaminetetraacetic acid (EDTA) before mixing with the lyophilized N_10_-C_22_ peptide (1.2 M excess of peptide). The conjugate was dialyzed overnight against 1X phosphate-buffered saline (PBS) and coupling was confirmed using SDS-PAGE analysis.

Thirty-seven overlapping 10-mer linear peptides were designed (Pepscan, Amsterdam, Netherlands) to span the SD1 region of DBPII (Table S1). Chemically Linked Peptides on Scaffolds (CLIPS) technology was used to synthesize the SD1_CLIPS_ peptide (C(T2-013)NYKRKRRERDWDCNT KKDVCIPDRRYQLC(T2-013)K(Aoa)), as previously described [19]. The conformation of SD1_CLIPS_ was constrained by first conjugating the outermost pair of cysteines (residues 1 and 30) using the T2-013 scaffold. The two interior cysteines (residues 14 and 21) were then deprotected and oxidized to form a disulfide bond. This peptide was conjugated to keyhole limpet hemocyanin (KLH) or bovine serum albumin (BSA) using a N-succinimidyl 4-formylbenzoate (S-4FB) linker via the C-terminal N-epsilon-aminooxyacetyl-L-lysine residue on the peptide (Pepscan, Amsterdam, Netherlands). Conjugation was monitored by adding 2-hydrazinpyridine to the carrier protein/peptide mixture. This reagent reacted with free S-4FB linkers on the carrier protein, producing a colored product, which allowed the reaction to be monitored over time. Excess peptide was removed by filtration. 

### 2.2. Animal Immunizations

For the (N_10_-C_22_)-diphtheria toxoid (DT) immunizations, five female BALB/c mice (6 to 8 weeks old) were immunized subcutaneously with (N_10_-C_22_)-DT or DT alone (30 µg/mouse) emulsified in Complete Freund’s Adjuvant (CFA) (catalogue no. F5881; Sigma, Oakville, Canada) on day 1. On days 21 and 31, mice were boosted with the immunogen (10 µg/mouse) emulsified in Incomplete Freund’s Adjuvant (IFA) (catalogue no. F5506; Sigma, Oakville, Canada) and the final sera samples were collected on day 45 via cardiac puncture. 

For the SD1_CLIPS_-KLH immunizations, five female BALB/c mice (6 to 8 weeks old) were immunized subcutaneously with SD1_CLIPS_-KLH or KLH alone (30 µg/mouse) emulsified in CFA on day 1. On days 21, 31, and 41, mice were boosted with the immunogen (10 µg/mouse on days 21 and 31, and 5 µg/mouse on day 41) emulsified in IFA. The final sera samples were collected on day 55 via cardiac puncture. All procedures were approved by the University of Alberta Animal Care and Use Committee (approval number: AUP00002124), and mice were handled in accordance with the Canadian Council on Animal Care Guidelines.

Rabbit antisera were generated commercially by ProSci Inc. (Poway, CA, USA). One rabbit was immunized with 160 µg of SD1_CLIPS_-BSA in CFA followed by three boosts with 80 µg in IFA on days 14, 28, and 43 after the first immunization. The final bleed was collected on day 56. 

### 2.3. ELISAs 

Indirect ELISAs were performed by coating 96-well plates (catalogue no. 439454; Thermo Fisher Scientific, Edmonton, Canada) with antigen diluted in 1X PBS, incubated overnight at 4 °C. Recombinant protein antigens were coated at 0.5 µg/mL, SD1ss was coated at 1.0 µg/mL, and all other synthetic peptides were coated at 5.0 µg/mL. Plates were blocked with 4% BSA (catalogue no. A7906; Sigma, Oakville, Canada) in 1X PBS for 1 h at 37 °C, then washed once with 1× PBST (0.1% Tween 20). Primary antibody samples were diluted in 2% BSA, added to wells, and incubated for 1 h at room temperature (RT). Plates were washed four times with 1× PBST, and 100 µL of horseradish peroxidase (HRP)-conjugated goat anti-mouse secondary antibody (1/3000) (catalogue no. 170-6516, Bio-Rad, Mississauga, Canada) was added to each well. After incubation for 1 h at RT, the plate was washed four times with 1X PBST, and 100 µL of 3,3′,5,5′-tetramethylbenzidine (TMB) (catalogue no. T0440; Sigma, Oakville, ON, Canada) was added to each well. After incubation at RT for 30 min, the reaction was stopped by adding an equal amount of H_2_SO_4_ (0.5 N) to each well, and the optical density (OD) of individual wells was read at 450 nm. All samples were run in duplicate and the mean OD for each antigen alone plus secondary was subtracted from the OD of each sample. 

Competition ELISAs were performed as above, except that primary antibodies were incubated with test peptides for 30 min at RT before addition to wells. To measure antibody avidity, ELISAs were performed using the same protocol for indirect ELISAs, except that 100 µL of 1 M NaSCN (catalogue no. 251410; Sigma, Oakville, Canada) or 1× PBS for control wells was added to each well following the primary antibody incubation. After a 10 min incubation at RT, the plates were washed four times with 1× PBST (0.1% Tween 20). The remaining steps were carried out as outlined for the indirect ELISA. Data represent samples tested in duplicate in at least two independent experiments.

The peptide library was screened by Pepscan-based ELISA (Pepscan, Amsterdam, The Netherlands). Briefly, the peptide array was incubated with 3D10 (0.2 µg/mL) overnight at 4 °C then washed with 1X PBST. The array was then incubated with HRP-conjugated rabbit anti-mouse IgG (catalogue no. 6175-05, Southern Biotech, Birmingham, AL, USA) and incubated for 1 h at 25 °C. After washing, 2,2′-azino-di-3-ethylbenzthiazoline sulfonate (ABTS) was added with 20 µL/mL of H_2_O_2_ (3%) and the color development was measured after 1 h. 

### 2.4. Statistical Analysis

Data were plotted using Prism software (version 8; GraphPad, San Diego, CA, USA). 

## 3. Results

### 3.1. N_10_-C_22_ Is the Minimal Epitope for 3D10 Recognition, but Does Not Elicit Cross-Reactive Antibodies

**Figure 2:**
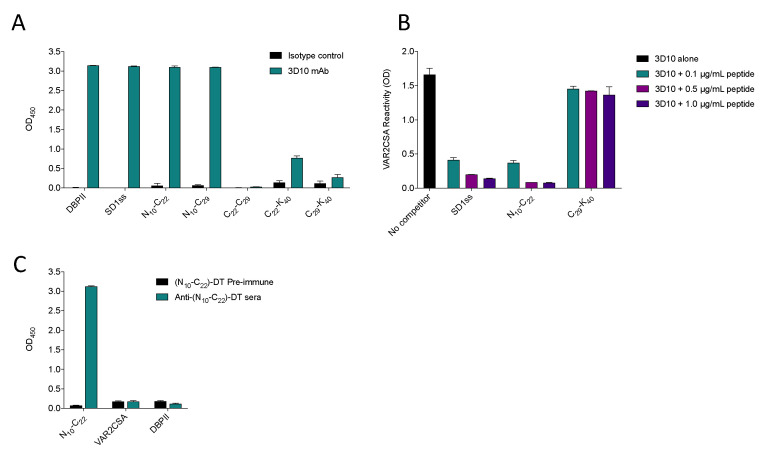
N_10_-C_22_ is the minimal epitope for 3D10 recognition of DBPII and VAR2CSA but does not elicit cross-reactive antibodies in mice. (**A**) Synthetic peptides spanning the SD1 region of DBPII were screened with 3D10 by ELISA. Recombinant DBPII protein was included as a positive control. (**B**) 3D10 was incubated alone (black bar) or with increasing concentrations of SD1ss, N_10_-C_22_, or C_29_-K_40_, then added to wells coated with recombinant full-length VAR2CSA. (**C**) Pooled sera from mice (n = 5) before and after immunization with (N_10_-C_22_)-DT were tested against N_10_-C_22_, VAR2CSA, and DBPII by ELISA. Data are mean ± standard deviation (SD).

In order to identify the minimal epitope for 3D10 recognition, we designed a number of synthetic peptides spanning the SD1 region of DBPII (Figure 1). In the native protein, this region contains four cysteine residues that form two disulfide bonds. We previously tried to recapitulate the native structure using a synthetic peptide, but based on mass spectrometry, the disulfide bonds did not form in the correct configuration (data not shown). Therefore, we designed a peptide called SD1ss, in which two of the cysteine residues were mutated to serines (‘ss’), which allowed us to control the intramolecular disulfide bonding pattern. When we screened these peptides by ELISA; the regions nearest the amino terminus of SD1 (N_10_-C_29_ and N_10_-C_22_) were strongly recognized by 3D10 (Figure 2A). The minimal epitope recognized by 3D10 was a 12 amino acid peptide called N_10_-C_22_ that was recognized with an endpoint titer of 2.7 ng/mL, similar to the reactivity against the parent protein DBPII. This finding is supported by previous reports that 3D10 recognizes the NXXRKR motif within this peptide [16,20]. Using a competition ELISA, this peptide was sufficient to block heterologous recognition of VAR2CSA by 3D10 (Figure 2B). When 3D10 was incubated with increasing concentrations of this peptide, N_10_-C_22_ blocked recognition of VAR2CSA to a similar degree as SD1ss. The C_29_-K_40_ peptide was included as a negative control for inhibition because 3D10 did not recognize this peptide (Figure 2A). Based on these results, we concluded that N_10_-C_22_ represents the minimal epitope for homologous 3D10 recognition of DBPII and mediates heterologous recognition of VAR2CSA.

We next immunized five BALB/c mice with the N_10_-C_22_ peptide conjugated to DT. The mice produced high levels of antibodies against N_10_-C_22_; however, the antibodies did not cross-react with VAR2CSA by ELISA (Figure 2C). This lack of cross-reactivity is not surprising, considering these antibodies also failed to recognize DBPII, the cognate protein from which the peptide was derived (Figure 2C). 

### 3.2. 3D10 Recognizes a Discontinuous Epitope in SD1

**Figure 3:**
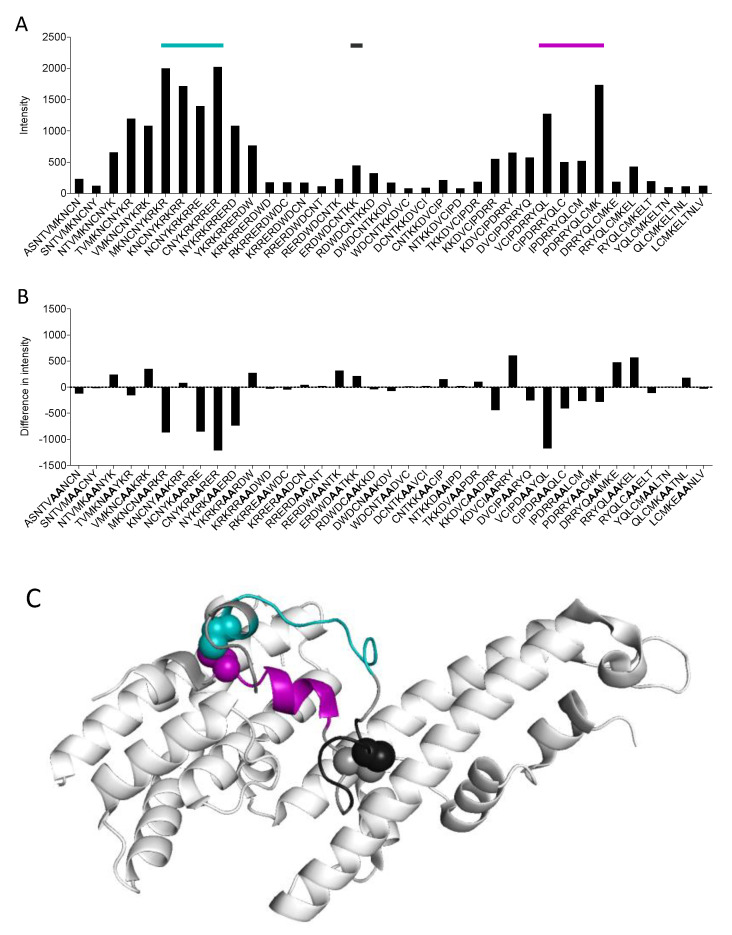
3D10 recognizes a discontinuous epitope in SD1. (**A**) An overlapping peptide array spanning the SD1 region of DBPII was screened with 3D10. The colors of the lines above the bar graph correspond to the region of SD1 mapped to the structure in (C). (**B**) The intensity of 3D10 recognition of peptides with alanine substitutions (bolded) was subtracted from that of the native sequence. Negative values indicate that 3D10 recognition was decreased following alanine substitution. (**C**) The structure of SD1 was determined from a published crystal structure of DBPII that was adapted using PyMOL (PDB ID 4NUU). The cysteines involved in disulfide bonds are shown as space-filling spheres.

To map the recognition site of 3D10, we designed an array of 37 overlapping linear peptides spanning SD1 of DBPII (Table S1). We used a Pepscan-based ELISA for this screen because the peptides can be coated at a high concentration, which allows for identification of minor parts of the epitope that may not be detected using a conventional ELISA. We screened this library with 3D10 and found that it recognized peptides from three regions of SD1 (Figure 3A). These results suggested that the epitope is discontinuous. We then screened a library of these peptides where two residues in each peptide were mutated to alanine (Figure 3B). Several of these mutations resulted in a significant decrease in 3D10 recognition. These data further confirm that sequences within the two outer segments in particular are critical for 3D10 recognition.

In the native DBPII structure, there are two disulfide bonds that bring these regions in close proximity, allowing them to form the discontinuous epitope (Figure 3C). This presents a significant challenge for vaccine development as conformational epitopes are notoriously difficult to recapitulate with synthetic peptides [21]. 

### 3.3. Immunization with SD1_CLIPS_ Elicited Cross-Reactive VAR2CSA Antibodies

**Figure 4:**
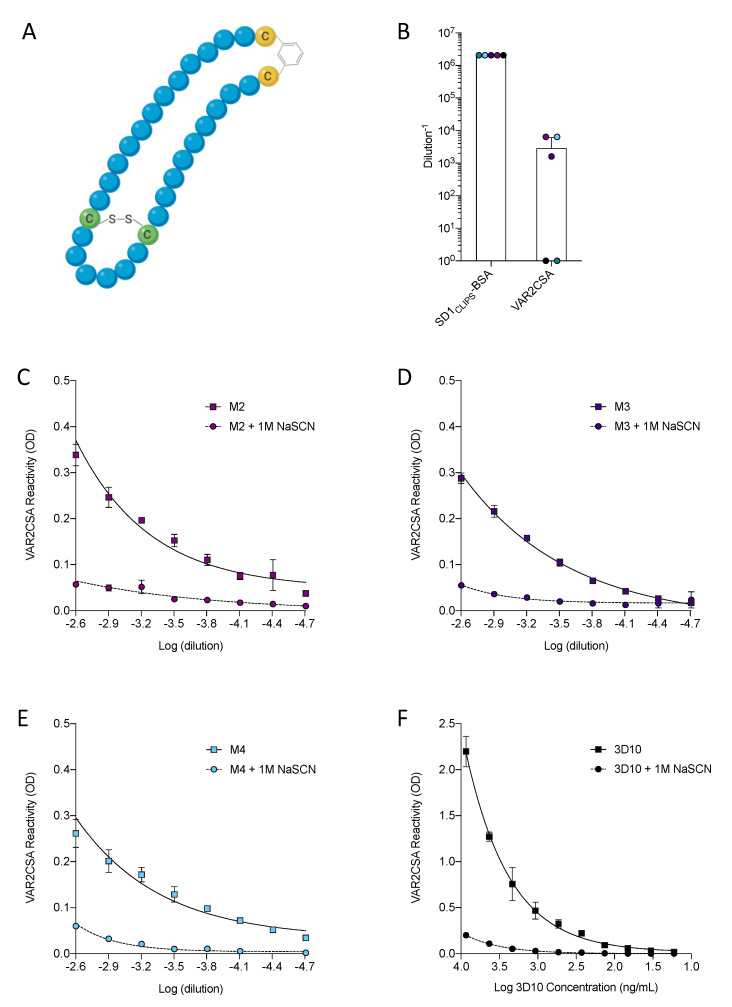
A conformationally constrained peptide encompassing the SD1 region of DBPII elicited antibodies that cross-react with VAR2CSA. (**A**) A synthetic 31 amino acid peptide of the SD1 region was designed using a chemical scaffold in place of one of the disulfide bonds. The cysteine residues that formed a disulfide bond are shown in green, and those involved in the chemical linkage are shown in yellow. (**B**) Endpoint titers for the individual mice immunized with SD1_CLIPS_-keyhole limpet hemocyanin (KLH) were measured against SD1_CLIPS_-bovine serum albumin (BSA) and VAR2CSA by ELISA. Data from each individual mouse (M#) are represented by a unique color. Endpoint titers were calculated relative to the mean optical density (OD) of the matching preimmune serum plus two standard deviations. (**C**–**F**) The avidity of the cross-reactive sera (**C–E**) and 3D10 (**F**) for VAR2CSA was determined by titrating the individual serum samples or 3D10 against VAR2CSA with and without the addition of 1 M NaSCN. Data are mean ± SD.

To recapitulate the immunogen that elicited the cross-reactive 3D10 mAb in DBPII, we designed a 31 amino acid peptide called SD1_CLIPS_ that uses CLIPS technology to control bond formation and conformationally constrain the peptide to its native structure. Specifically, a synthetic scaffold was inserted in place of one of the disulfide bonds to ensure that the disulfide bond formed between the correct pair of cysteines (Figure 4A). After confirming that 3D10 recognized this peptide by ELISA (endpoint titer: 20.5 ng/mL), we immunized five BALB/c mice with SD1_CLIPS_ conjugated to keyhole limpet hemocyanin (KLH). All mice produced high levels of antibodies against SD1_CLIPS_ and 60% (3/5) of the mice also made antibodies that cross-reacted with VAR2CSA (Figure 4B). Although the titers of the anti-VAR2CSA antibodies were relatively low compared to those against SD1_CLIPS_, these results demonstrate that a peptide immunogen successfully recapitulated a cross-reactive conformational epitope.

When we measured the avidity of the cross-reactive antibodies against VAR2CSA, the avidity was low compared to the avidity of the antibodies for their immunogen. Incubation with 1 M NaSCN significantly reduced antibody binding to VAR2CSA across a range of dilutions (Figure 4C–E), whereas there was little reduction in antibody binding to SD1_CLIPS_ (Figure S1A–E). It is not uncommon for cross-reactive antibodies to have a lower avidity for the heterologous antigen. However, this does not negate the potential functional relevance of these antibodies. In fact, the avidity of these antibodies for VAR2CSA is similar to that of 3D10 (Figure 4F), which blocked VAR2CSA-expressing *P. falciparum* parasites from binding to the placental ligand in vitro [9].

### 3.4. Fine Specificity and Avidity of Anti-SD1_CLIPS_ Antibodies in Individual Mice

**Figure 5:**
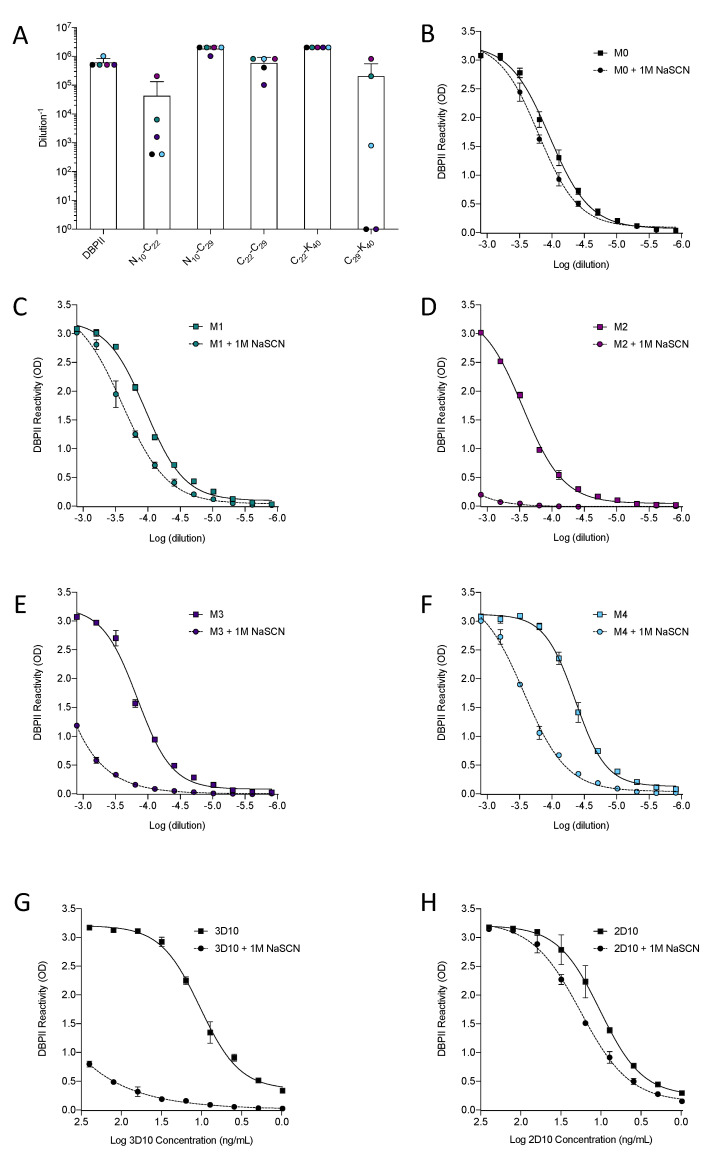
Cross-reactive sera that recognized VAR2CSA had low avidity against DBPII. (**A**) Endpoint titers for the individual mice immunized with SD1_CLIPS_-KLH were measured against DBPII and the synthetic peptides spanning the SD1 region. Endpoint titers were calculated relative to the mean OD of the matching preimmune serum plus two standard deviations. Data from each individual mouse (M#) are represented by a unique color. (**B**–**F**) The avidity of the individual serum samples for DBPII was determined by titrating against DBPII with and without the addition of 1 M NaSCN. (**G**–**H**) The avidity of 3D10 and 2D10 (a non-cross-reactive DBPII monoclonal antibody) against DBPII was determined by titrating the monoclonal antibodies against DBPII with or without the addition of 1 M NaSCN. Data are mean ± SD.

Despite making similar levels of antibodies against the SD1_CLIPS_ immunogen, only three out of the five mice made cross-reactive antibodies that recognized VAR2CSA. This prompted us to evaluate the fine specificity of the individual immune responses of each mouse for DBPII and the smaller peptides within the SD1 region. Sera from all of the mice recognized DBPII, N_10_-C_29_, C_22_-K_40_, and C_22_-C_29_ at similarly high levels (Figure 5A). However, the mice had varying levels of antibodies against the shorter N_10_-C_22_ and C_29_-K_40_ peptides. For instance, serum from mouse M0 (black circles in Figure 5A) had an endpoint titer of 1/400 against N_10_-C_22_ and did not recognize C_29_-K_40_, whereas serum from mouse M2 (light purple circles in Figure 5A) had an endpoint titer of over 1/200,000 against N_10_- C_22_ and over 1/800,000 against C_29_-K_40_. Despite being of the same inbred strain of mouse, each of the five mice had a unique immune response to different regions of the SD1_CLIPS_ immunogen.

There was no discernable difference in the recognition patterns of sera that cross-reacted with VAR2CSA (mice M2, M3, and M4) and sera that did not (mice M0 and M1). However, the cross-reactive sera had much lower avidity for DBPII than the non-cross-reactive sera (Figure 5B–F). This result is consistent with the avidity of 3D10 for DBPII, which is significantly lower compared to another DBPII monoclonal antibody, 2D10, which we showed previously does not cross-react with VAR2CSA (Figure 5G,H) [9]. Together, these results suggest that antibodies against DBPII that cross-react with VAR2CSA exhibit low avidity against both of these proteins.

### 3.5. SD1_CLIPS_ Elicits Cross-Reactive Antibodies in a Rabbit

**Figure 6:**
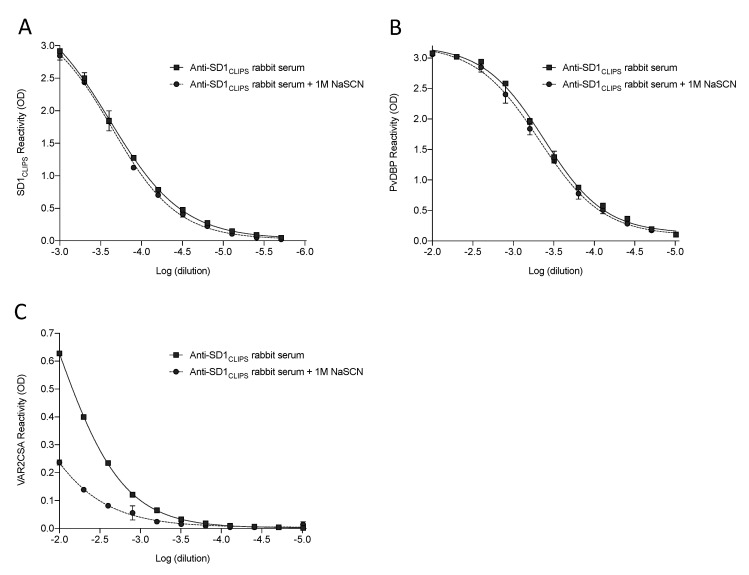
SD1_CLIPS_ elicits cross-reactive antibodies in a rabbit. The avidity of the anti-SD1_CLIPS_ rabbit serum against (**A**) SD1_CLIPS_, (**B**) DBPII, and (**C**) VAR2CSA was determined by titrating the serum against the antigen with and without the addition of 1 M NaSCN. Data are mean ± SD.

To determine if antibodies cross-reactive with VAR2CSA can be elicited in a different animal, we immunized a rabbit with the same SD1_CLIPS_ peptide, only conjugated to BSA. This conjugate was highly immunogenic in the rabbit, with an endpoint titer against the peptide of 1/256,000 and very high avidity (Figure 6A). These antibodies also strongly recognized DBPII and, unlike the mouse serum, their avidity for DBPII was high (Figure 6B). Importantly, the serum also recognized VAR2CSA (Figure 6C), and consistent with the results from mice, these antibodies were not as strong (endpoint titer of 1/1600) and were of low avidity. 

## 4. Discussion

In this study, we used an epitope-focused vaccine to enhance the intrinsic cross-reactivity that exists between DBPII and VAR2CSA. We identified a conformationally constrained synthetic peptide that elicited an antibody response against not only its parent protein, DBPII, but also against a heterologous antigen, VAR2CSA. This immunogen was based on the recognition site of a monoclonal antibody that is cross-reactive against VAR2CSA [9]. We initially mapped the minimal epitope to a 12 amino acid linear peptide within SD1. However, immunization with this peptide failed to elicit antibodies to DBPII and, not surprisingly, the antibodies did not cross-react with VAR2CSA. These results suggest that the linear N_10_-C_22_ peptide is sufficient as an antigen for 3D10 recognition but is not a suitable immunogen to generate antibodies to the desired epitope in DBPII. It is also possible that immunization with this peptide elicited antibodies that recognized an unnatural epitope formed by the synthetic peptide that is not present in DBPII. Consistent with our results, others reported that 3D10 recognized a peptide from the same region of DBPII that overlapped with N_10_-C_22_ by seven amino acids [16]. However, this peptide also failed to elicit anti-DBPII antibodies. 

The results of our peptide library screen expanded the minimal epitope to encompass three distinct segments of SD1 that are discontinuous yet constrained in the native protein by two disulfide bonds. To recapitulate this epitope, we designed the SD1_CLIPS_ peptide that contains all regions of the discontinuous epitope and replaced one of the disulfide bonds with a synthetic scaffold. This allowed for control over bond formation in the peptide, constraining the bonding pattern to the structure in the parent protein. With this approach, we succeeded in generating the discontinuous epitope as it was in DBPII, overcoming a common challenge in peptide vaccine development. 

While all mice immunized with SD1_CLIPS_-KLH had high endpoint titers against the immunogen (over 1/2.0 million), only three out of five mice made cross-reactive antibodies that recognized VAR2CSA. To better understand this, we evaluated the immune responses of the five mice to peptides from different regions of SD1 and found that no two mice had the same recognition profile. Certain peptides were recognized very strongly (endpoint titers over 1/1.0 million) by sera from some mice but not others, revealing significant variation in the fine specificity of immune responses among the five mice. However, there was no discernable difference in the recognition patterns of the SD1 peptides between the sera samples that cross-reacted with VAR2CSA and those that did not. 

In both the mice and the rabbit, the cross-reactive antibodies had low avidity for VAR2CSA. These experiments highlight a common feature of cross-reactive antibodies that lower avidity antibodies may be multispecific for similar but not identical epitopes in different antigens [21]. There is evidence that people living in areas of high malaria transmission intensity develop lower avidity antibodies to some *P. falciparum* antigens, compared to those living in areas of low transmission intensity who develop relatively low titers of higher avidity antibodies [22,23]. This has been attributed to increased exposure to a highly diverse population of parasites in high transmission settings [22,24] and/or impaired affinity maturation as a result of infection [25,26,27]. Alternatively, lower avidity antibodies may reflect an adaptive advantage that allows the immune system to respond to antigens from different *P. falciparum* strains, as has been suggested for other pathogens [28]. However, several studies demonstrated that higher avidity antibodies against blood stage *P. falciparum* antigens provided greater protection from severe [29] or clinical malaria [30]. Importantly, in a retrospective case-control study and a longitudinal study in Cameroon, higher avidity anti-VAR2CSA antibodies were associated with protection from malaria in pregnancy [31,32]. Thus, it is essential to explore ways to increase the avidity of these cross-reactive antibodies against VAR2CSA. Indeed, the low-avidity 3D10 antibody can block parasite binding to chondroitin sulfate A (CSA), but only when used at a high concentration that is unlikely to be elicited through immunization [9]. 

In order to further develop this immunogen as a vaccine candidate, it is crucial to increase not only the avidity of the antibodies for VAR2CSA, but also the titers. This will facilitate the evaluation of the functional activity of these cross-reactive antibodies, since a high concentration of anti-VAR2CSA antibodies is required to block parasite adhesion to CSA in vitro [9,10,33,34]. We will use different methods to increase both the titer and avidity of the cross-reactive antibodies. For instance, we will employ different boosting schedules and/or adjuvant strategies that have been shown to increase the avidity of some antibody responses [35,36]. We will also further investigate the VAR2CSA target of these cross-reactive antibodies to inform optimal peptide design. 

## 5. Conclusions

Ultimately, our goal is to exploit the cross-reactive epitope in PvDBP that generates antibodies to shared epitopes in VAR2CSA. The structural peptide reported here provides a template for the synthesis of a discontinuous conformational epitope from PvDBP that elicits cross-reactive antibodies to VAR2CSA. With further optimization to enhance immunogenicity and avidity, this peptide could be developed as a new vaccine candidate against placental malaria.

## Supplementary Materials

The following are available online at https://www.mdpi.com/2076-393X/8/3/392/s1. Table S1: Peptides used for the Pepscan-based ELISA screen in Figure 3A,B. Figure S1: The avidity of individual mouse sera against SD1_CLIPS_-BSA.
