# Supplementary material for: Generation of a Peptide Vaccine Candidate against Falciparum Placental Malaria Based on a Discontinuous Epitope
Source: Vaccines (Basel). 2020 Jul 18;8(3):392. doi: 10.3390/vaccines8030392 (PMC7564767; doi:10.3390/vaccines8030392)
Supplement: Supplementary file 1 [file vaccines-08-00392-s001.pdf]

vaccines

Table S1. Peptides used for the Pepscan-based ELISA screen in Figure 3A and B.

Start (residue no.)	End (residue no.)	Native sequence	Mutated sequence
1	10	ASNTVMKNCN	ASNTV**AA**NCN
2	11	SNTVMKNCNY	SNTVM**AA**CNY
3	12	NTVMKNCNYK	NTVMK**AA**NYK
4	13	TVMKNCNYKR	TVMKN**AA**YKR
5	14	VMKNCNYKRK	VMKNC**AA**KRK
6	15	MKNCNYKRKR	MKNCN**AA**RKR
7	16	KNCNYKRKRR	KNCNY**AA**KRR
8	17	NCNYKRKRRE	NCNYK**AA**RRE
9	18	CNYKRKRRER	CNYKR**AA**RER
10	19	NYKRKRRERD	NYKRK**AA**ERD
11	20	YKRKRRERDW	YKRKR**AA**RDW
12	21	KRKRRERDWD	KRKRR**AA**DWD
13	22	RKRRERDWDC	RKRRE**AA**WDC
14	23	KRRERDWDCN	KRRER**AA**DCN
15	24	RRERDWDCNT	RRERD**AA**CNT
16	25	RERDWDCNTK	RERDW**AA**NTK
17	26	ERDWDCNTKK	ERDWD**AA**TKK
18	27	RDWDCNTKKD	RDWDC**AA**KKD
19	28	DWDCNTKKDV	DWDCN**AA**KDV
20	29	WDCNTKKDVC	WDCNT**AA**DVC
21	30	DCNTKKDVCI	DCNTK**AA**VCI
22	31	CNTKKDVCIP	CNTKK**AA**CIP
23	32	NTKKDVCIPD	NTKKD**AA**IPD
24	33	TKKDVCIPDR	TKKDV**AA**PDR
25	34	KKDVCIPDRR	KKDVC**AA**DRR
26	35	KDVCIPDRRY	KDVCI**AA**RRY
27	36	DVCIPDRRYQ	DVCIP**AA**RYQ
28	37	VCIPDRRYQL	VCIPD**AA**YQL
29	38	CIPDRRYQLC	CIPDR**AA**QLC
30	39	IPDRRYQLCM	IPDRR**AA**LCM
31	40	PDRRYQLCMK	PDRRY**AA**CMK
32	41	DRRYQLCMKE	DRRYQ**AA**MKE
33	42	RRYQLCMKEL	RRYQL**AA**KEL
34	43	RYQLCMKELT	RYQLC**AA**ELT
35	44	YQLCMKELTN	YQLCM**AA**LTN
36	45	QLCMKELTNL	QLCMK**AA**TNL
37	46	LCMKELTNLV	LCMKE**AA**NLV

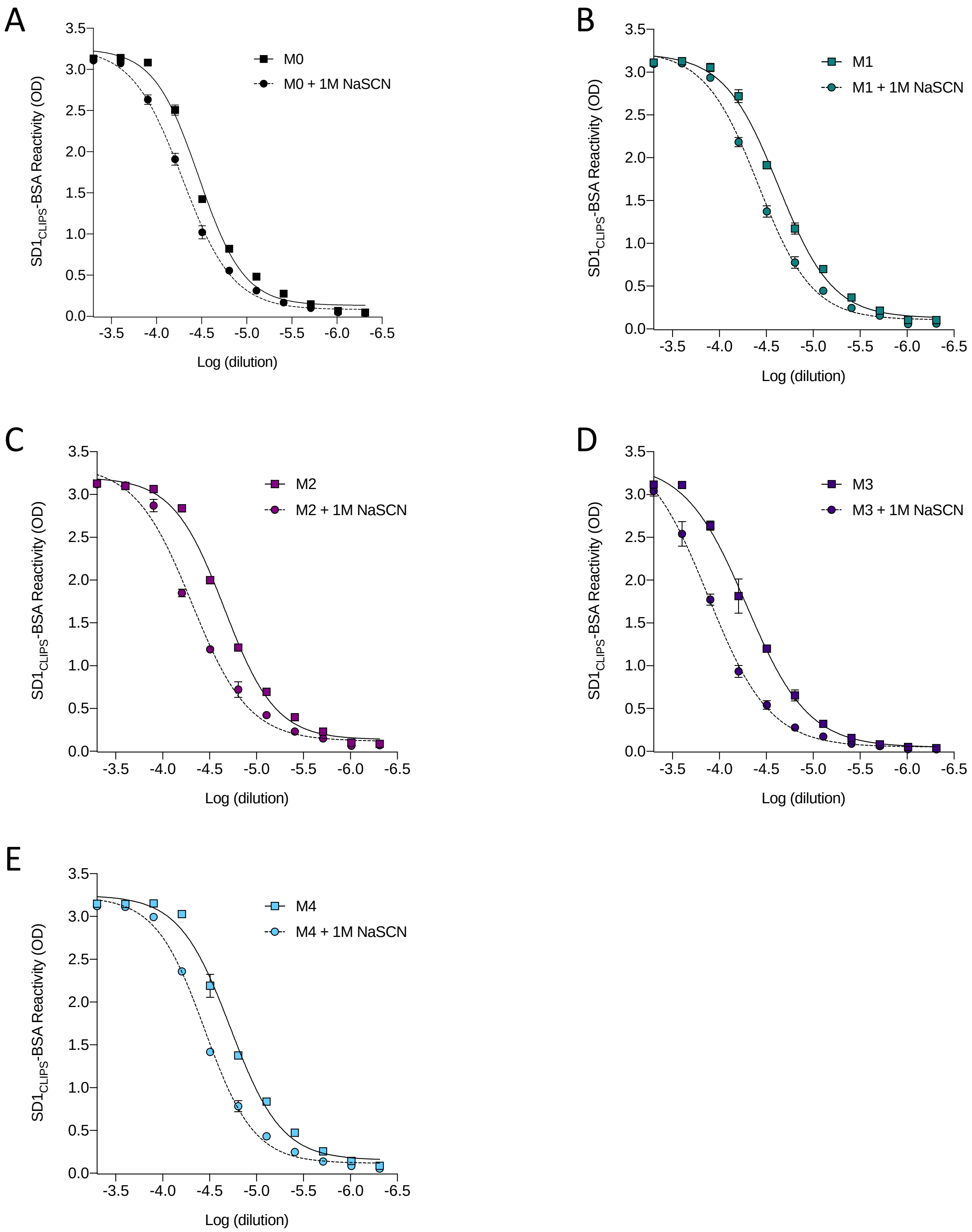

Figure S1. The avidity of individual mouse serum against SD1CLIPS-BSA. (A-E) The avidity of individual serum samples for SD1CLIPS-BSA was determined by titrating serum against this antigen with and without the addition of 1M NaSCN. Data are mean ± SD.
